# Two orthogonal cleavages separate subunit RNAs in mouse ribosome biogenesis

**DOI:** 10.1093/nar/gku787

**Published:** 2014-09-04

**Authors:** Minshi Wang, Leonid Anikin, Dimitri G. Pestov

**Affiliations:** 1 Department of Cell Biology, Rowan University School of Osteopathic Medicine, Stratford, NJ 08084, USA; 2 Graduate School of Biomedical Sciences, Rowan University, Stratford, NJ 08084, USA

## Abstract

Ribosome biogenesis is a dynamic multistep process, many features of which are still incompletely documented. Here, we show that changes in this pathway can be captured and annotated by means of a graphic set of pre-rRNA ratios, a technique we call Ratio Analysis of Multiple Precursors (RAMP). We find that knocking down a ribosome synthesis factor produces a characteristic RAMP profile that exhibits consistency across a range of depletion levels. This facilitates the inference of affected steps and simplifies comparative analysis. We applied RAMP to examine how endonucleolytic cleavages of the mouse pre-rRNA transcript in the internal transcribed spacer 1 (ITS1) are affected by depletion of factors required for maturation of the small ribosomal subunit (Rcl1, Fcf1/Utp24, Utp23) and the large subunit (Pes1, Nog1). The data suggest that completion of early maturation in a subunit triggers its release from the common pre-rRNA transcript by stimulating cleavage at the proximal site in ITS1. We also find that splitting of pre-rRNA in the 3′ region of ITS1 is prevalent in adult mouse tissues and quiescent cells, as it is in human cells. We propose a model for subunit separation during mammalian ribosome synthesis and discuss its implications for understanding pre-rRNA processing pathways.

## INTRODUCTION

**Figure 1.**
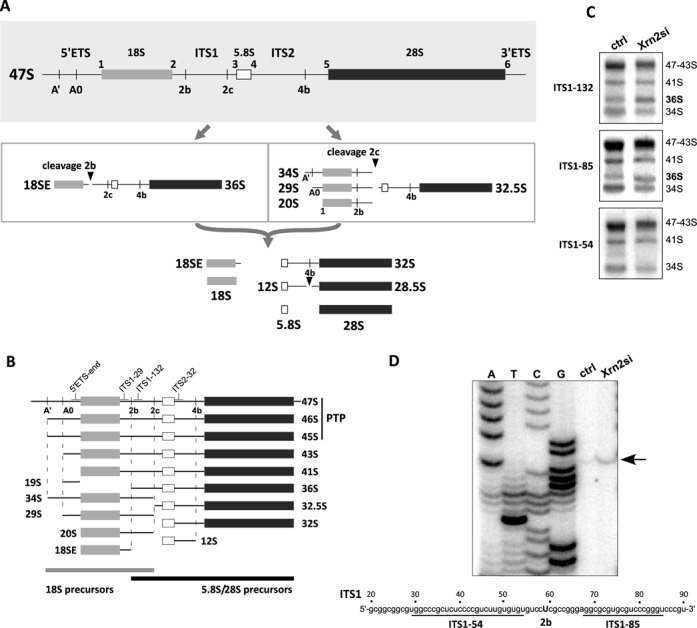
Mapping of the ITS1 cleavage sites in mouse pre-rRNA. (A) Major processing sites in the mouse 47S pre-rRNA transcript are shown at the top. Separation of subunit RNAs can occur through ITS1 cleavages at sites 2b or 2c, giving rise to distinct sets of intermediates (boxed areas). After separation in ITS1, SSU and LSU precursors continue maturation to 18S and 5.8S/28S rRNAs. Early intermediates are omitted for clarity; a more detailed diagram of the mouse pre-rRNA processing pathway is shown in Supplementary Figure S2. (B) Structure of processing intermediates discussed in the text. Relative positions of hybridization probes are indicated. PTP, ‘primary transcript plus’, a combination of three early pre-rRNAs used in calculating precursor ratios. (C) Northern hybridizations of pre-rRNA in cells transfected with non-targeting siRNA (ctrl) or siRNA against Xrn2. Probes are indicated on the left. (D) Primer extension to determine the precise location of the 2b cleavage. Arrow indicates a stop corresponding to the ‘U’ at position +59 relative to the start of mouse ITS1 (Genbank reference sequence X82564). Two probes used in hybridizations to confirm the cleavage site (panel C) are underlined in the nucleotide sequence.

The eukaryotic ribosome is made of four ribosomal RNAs (rRNAs) and around 80 ribosomal proteins. Three of the rRNAs, 18S, 5.8S and 28S (25S in yeast), are transcribed by Pol I as a single precursor (pre-rRNA) that contains several transcribed spacers in addition to the mature rRNA sequences. In mammals, there are typically four spacers designated 5′ETS, ITS1, ITS2 and 3′ETS, which are removed from the pre-rRNA transcript in the course of its post-transcriptional maturation (1). The removal of spacers is coupled with the incorporation of ribosomal proteins and assembly of the 3D ribosome structure. An important step in pre-rRNA processing is the endonucleolytic separation of the primary transcript within ITS1 (Figure 1A). After the separation, the two parts of the transcript continue their maturation in a largely independent manner to form 18S rRNA in the small subunit (SSU) and 5.8S, 28S in the large subunit (LSU). Processing of pre-rRNA and assembly of the nascent subunits requires >200 transiently associating assembly factors that include enzymes (such as RNA helicases and ribonucleases) and proteins with non-enzymatic functions (2,3).

In the past two decades, ribosome biogenesis has been studied extensively using *Saccharomyces cerevisiae* as a model organism. As a fundamental biosynthetic activity, ribosome biogenesis is expected to be highly conserved across species. Indeed, recent studies have demonstrated that a number of ribosome synthesis factors in higher eukaryotes perform functions similar to their counterparts in yeast (4–7). However, it has also become clear that because of the vast evolutionary distances separating yeast and mammals, ribosome synthesis in mammalian species does not always follow the yeast ‘blueprint’. Studies of ribosome synthesis in higher organisms revealed small nucleolar RNAs and proteins that do not have a yeast homolog (8–11). In some cases, even homologous ribosome synthesis factors have diverged in their function; for example, yeast Nip7 and Spb1 are required for maturation of 5.8S and 25S rRNAs, whereas their human homologs NIP7 and FTSJ3 are involved in 18S rRNA synthesis (12). Some general features of pre-rRNA processing differ between yeast and mammals as well. Whereas 70–80% of nascent pre-rRNA transcripts undergo cotranscriptional ITS1 cleavage in rapidly growing *S. cerevisiae* cells, the primary transcript is rarely, if ever, split cotranscriptionally in mammalian cells (13,14). Recent studies addressing ITS1 processing in human cell lines have shown that it is more complicated than in yeast cells and involves both endo- and exonucleolytic activities (15–17).

In the early processing of pre-rRNA leading up to the separation of the SSU and LSU precursors, parallel and alternative routes exist (Supplementary Figures S1 and S2), which complicates analysis of these processing steps. Moreover, non-coding spacers in pre-rRNA diverge rapidly in evolution and exhibit few conserved elements (18), making ITS1 cleavage sites difficult or impossible to predict even for closely related species. We reasoned that analysis in the mouse, when combined with human data, would help to better understand the conserved features of mammalian subunit pre-rRNA separation. In this study, we completed mapping of the two major cleavage sites (termed 2b and 2c) in mouse ITS1 and examined how these cleavages were affected by depletion of several factors involved in either SSU or LSU maturation. To improve our ability to deconstruct changes in pre-rRNA processing, we employed an approach termed RAMP (Ratio Analysis of Multiple Precursors), in which quantitative measurements of pre-rRNA levels are converted into an array of ratios presented in a graphical format. Using RAMP, we probed the effects of downregulating the expression of mouse proteins Rcl1, Fcf1/Utp24 and Utp23, whose human and yeast counterparts function in 18S rRNA maturation in the SSU (17,19–24). To assess the effects of blocking 5.8S/28S rRNA maturation, we knocked down LSU assembly factors Pes1 and Nog1 (4,25,26). Based on results of this analysis and the data from previous studies addressing mouse and human pre-rRNA processing, we propose a new unified model for the separation of the small and LSU RNAs during mammalian ribosome biogenesis.

## MATERIALS AND METHODS

### Cell culture

NIH and Balb/c 3T3 cells were cultured as previously described (27). Mouse embryonic fibroblasts generated from 129/SvE mice were analyzed at passage 2. J1 embryonic stem cells were cultured in feeder-free condition.

### RNA interference

Plasmids and siRNAs were delivered to cells via calcium phosphate transfection as reported earlier (28). For siRNA-mediated knockdowns, we used siGENOME SMARTpools (Dharmacon). For shRNA-mediated knockdowns, we constructed a new vector from pTRIPz (Open Biosystems) by transferring a 5224-bp fragment excised with Pst I into the pUC19 vector linearized with Pst I. shRNA cassettes were recloned from the mouse pGIPz library (Open Biosystems) into the resulting plasmid, termed pIVRE, through Mlu I and Xho I restriction sites. Cells stably transfected with pIVRE constructs were selected with puromycin and single-cell clones were obtained from the transfected pool. Expression of the shRNAs was induced with 2 μg/ml doxycycline for 72 h. To assay for gene knockdown efficiency, reverse transcription was performed using ReverseAid Reverse Transcriptase (Fermentas), cDNA was diluted 1:5 with water and amplified using SYBR FAST Universal qPCR Master Mix (KAPA Biosystems) on Mastercycler ep realplex (Eppendorf). Primers for quantitative polymerase chain reaction (qPCR) are listed in Supplementary Table S1. The remaining mRNA level was estimated by normalization of the target mRNA level to that of ribosomal protein Rpl23.

### RNA analysis

Total cellular RNA was extracted using TRI reagent or RNAzol RT (Molecular Research Center, Inc) according to the manufacturer's protocols. Note that 2–3 μg of total RNA per lane was resolved on 1% agarose gels (29) and analyzed by northern hybridizations with ^32^P-labeled oligonucleotide probes (Supplementary Table S1) as described (30). Primer extension was performed as described previously (31) using primer ITS1–184 (Supplementary Table S1).

### Ratio analysis of multiple precursors

**Figure 2.**
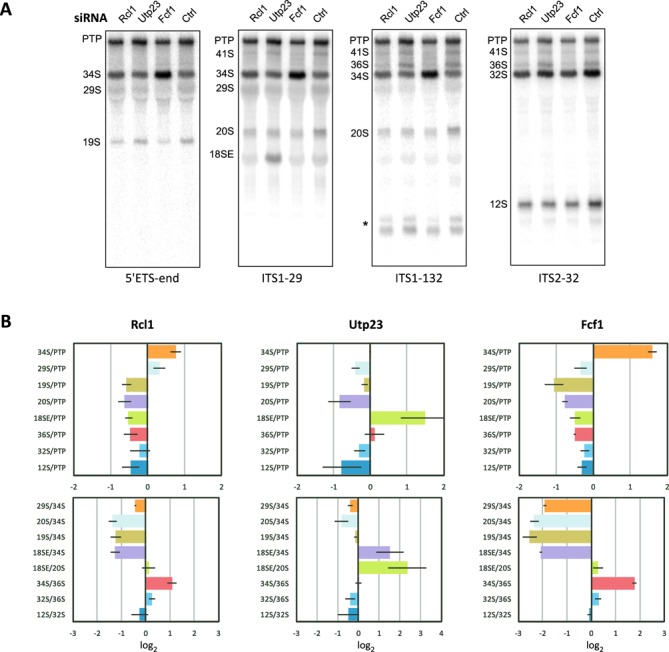
Effects of knockdowns of SSU synthesis factors on mouse pre-rRNA processing. (A) Northern hybridizations of RNA isolated from cells after siRNA-mediated depletion of Rcl1, Utp23 and Fcf1 or after transfection with non-targeting siRNA (Ctrl). Probes used for each hybridization are indicated at the bottom. Images for a representative transfection, corresponding to Experiment 2 in Supplementary Figure S4, are shown. PTP designates the combination of the closely migrating 47S, 46S and 45S pre-rRNAs, which are detectable with each probe. Asterisk, the internal ITS1 fragment released by the 2b and 2c cleavages and further trimmed by exonucleases (31). (B) RAMP profiles for the knockdowns. Each profile shows log_2_ ratios of precursors normalized to non-targeting siRNA control (see Materials and Methods for details). The profiles in this figure show log average ± standard deviation for three independent siRNA transfections. Individual RAMP profiles for the repeats of the transfections are shown in Supplementary Figure S4B and C. Ratios were derived using the same probes as those indicated in panel A.

To generate a RAMP profile, we begin by carrying out a series of northern hybridizations with the knockdown and control (non-silenced) samples and quantify steady-state levels of various rRNA precursors by phosphorimaging. Next, we calculate precursor ratios for each sample (34S/36S pre-rRNAs, 12S/32S, etc.). It is important that each ratio is derived from one lane in the same hybridization to avoid artifacts arising from uneven sample loading and variations in hybridization efficiency. In the next step, the log_2_ values of each precursor ratio in the reference sample (transfected with non-silencing control siRNA) are subtracted from the corresponding log_2_ ratios in the knockdown of the gene of interest. The resulting normalized log ratios for the knockdown are graphed as color-coded bars arranged in a predetermined order (Figure 2B).

We find that combining three types of ratios in a single RAMP profile is informative. One is the ratio of a pre-rRNA level relative to that of the primary transcript (Figure 2B, shown in upper panels). Because the full-length transcript (47S pre-rRNA) is rapidly converted to 46S and 45S pre-rRNA and these three species cannot be separated on a regular agarose gel, we use their total hybridization signal, referred to as the ‘primary transcript plus’ (PTP), in all calculations. The second type of ratio includes pre-rRNAs that are in the immediate substrate–product relationship, such as 18SE/20S and 12S/32S. The third type is the ratio of mutually exclusive intermediates, such as 34S/36S (Figure 2B, lower panels). To make it easier to visualize and compare patterns in RAMP profiles, we typically arrange data in two separate charts, or ‘tiles’, per knockdown (note that all ratios must be labeled and arrayed consistently for all RAMP profiles being compared).

**Figure 3.**
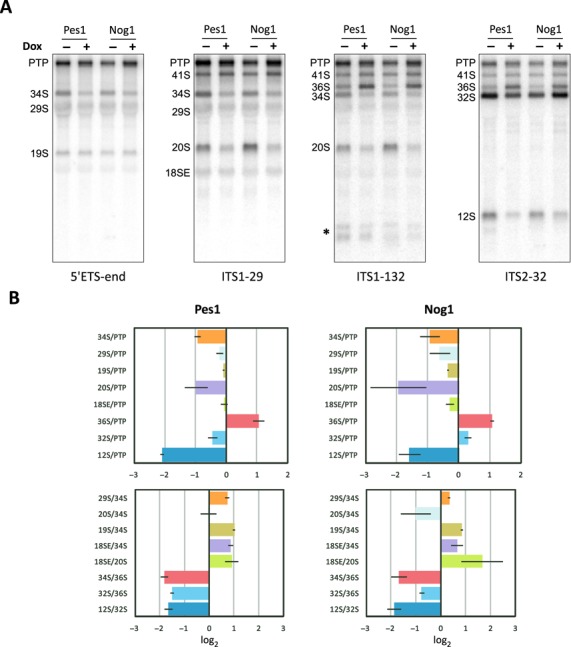
Effects of knockdowns of LSU synthesis factors on mouse pre-rRNA processing. (A) Northern hybridizations of RNA isolated from cells expressing doxycycline-inducible shRNAs that downregulate Pes1 and Nog1. Knockdown efficiency is shown in Supplementary Figure S5A; images are from Experiment 2. A control doxycycline treatment of cells transfected with an empty vector showed no pre-rRNA processing defects (Supplementary Figure S5B). Probes used for each hybridization are indicated at the bottom. PTP designates the combined 47S, 46S and 45S pre-rRNAs, which migrate close on the gel. Asterisk, exonuclease-processed products of ITS1 cleavages. (B) RAMP profiles for the knockdowns. Profiles show the log_2_ ratios of precursors normalized to non-induced control (see Materials and Methods for details). The log average ± standard deviation for each ratio are from three knockdown experiments (Supplementary Figure S5A). Ratios were derived using the same probes as those indicated in panel A.

All ratios for an individual RAMP profile should be generated from the same pair of biological samples (knockdown and reference) because fluctuations in pre-rRNA transcription, which is highly responsive to the physiological state of cells, can be a significant source of noise in this assay. As illustrated by data in this paper, visually comparing RAMP profiles for experimental repeats of a factor knockdown can be a useful approach to identify changes in processing that are independent on the factor's depletion level. Another practical way to assess reliability of the observed processing changes and robustness of the entire pattern is to plot average and standard deviation for log ratios from independent experimental repeats, as shown in Figures 2B and 3B. To ensure reproducibility, RAMP profiles in this study were generated for at least three independent experiments for each knockdown using different amounts of siRNA pools in each transfection.

## RESULTS

### Mapping of major cleavage sites in mouse ITS1

Our previous work has shown that the separation of the SSU and LSU rRNAs in the primary pre-rRNA transcript in mouse cells involves two cleavage events at sites termed 2b and 2c (Figure 1A). Cleavage at site 2c occurs at positions +837/+857 in the mouse ITS1 sequence and generates 32.5S pre-rRNA, a 5.8S/28S rRNA precursor containing ∼150 nt of the ITS1 sequence at its 5′ end. This extension is rapidly removed by the exonuclease Xrn2 (31). In addition to 32.5S pre-rRNA, cleavage at site 2c also generates an assortment of 18S rRNA precursors with various 5′ ends, termed 34S, 29S and 20S pre-rRNAs (Figure 1A and B).

Cleavage at site 2b in the 5′ terminal region of ITS1 generates 36S pre-rRNA (Figure 1A), which is also stabilized by Xrn2 depletion (31) (Figure 1C). Since the precise position of site 2b has not been previously determined, we performed primer extensions on total RNA isolated from the wild-type and Xrn2-depleted cells. This assay revealed a single primer extension stop corresponding to cleavage after position +58 from the beginning of ITS1; the intensity of this stop increased after Xrn2 depletion (Figure 1D). To confirm the location of site 2b, we designed hybridization probes flanking the position identified by primer extension (Figure 1D). As expected, only the 3′ probe ITS1–85 was able to detect the 36S pre-rRNA, while the probe ITS1–54 located 5′ to site 2b did not hybridize with this precursor (Figure 1C). The identified location of site 2b is consistent with early S1 nuclease mapping studies, in which rapid cleavage of the mouse pre-rRNA transcript was reported to occur ∼55 nt downstream from the 3′ end of the 18S rRNA sequence (32). Cleavage at site 2b generates an 18S precursor that we will refer to as 18SE pre-rRNA (extended) for consistency with the equivalent human 18S-E species, formed by cleavage at nucleotides +78/+81 in human ITS1 (16). The mouse and human cleavage sites are located 5′ to highly G+C-rich regions that can potentially fold into multiple alternative secondary structures, but there is no apparent sequence homology between the sites. The latter observation was not completely unexpected given the high variability of the first half of ITS1 in eukaryotic pre-rRNA (18).

### RAMP, a graphical approach to study complex processing steps

The existence of both 18S and 5.8S/28S precursors that have extensions spanning the 2b–2c region of ITS1 (Figure 1B) implies that the two ITS1 sites are not cleaved simultaneously. To investigate how processing of the SSU and LSU parts of the pre-rRNA transcript influences subunit RNA separation, we examined the effects of RNAi-mediated depletion of mouse proteins that have conserved yeast and human homologs required for SSU and LSU synthesis. Multiple pre-rRNA levels changed after the knockdowns, as was evident from northern hybridizations (Figures 2A and 3A). To reliably identify changes in the pathway, some way of integrating information on multiple pre-rRNAs was therefore necessary. One previously used approach in mammalian pre-rRNA analysis was to collate data on precursor levels in cells with the knockdown of a gene of interest relative to control cells (e.g. those transfected with scrambled siRNA), displaying normalized levels as a heatmap. This approach was successfully used for clustering ribosome synthesis factors into classes based on similarity of their pre-rRNA processing patterns (11,33). However, information on precursor levels alone often leaves considerable ambiguity with regard to which steps in the pathway are affected (for example, a decrease in a pre-rRNA may be due to a direct block in its formation, reduced level of one of its precursors or accelerated conversion to a downstream intermediate). How much pre-rRNA levels change relative to control cells also strongly depends on the extent of depletion achieved in each knockdown experiment. This complicates comparison of separate experiments, especially if the factors being studied are depleted to different levels.

We reasoned that instead of precursor levels, the state of the pre-rRNA processing pathway could be alternatively described through a set of ratios representing quantitative relationships between its various components. With this in mind, we developed a data analysis workflow in which measurements of pre-rRNA levels are converted into a series of ratios arrayed in a compact color-coded logarithmic bar-graph form we called a ‘RAMP profile’ (Figures 2B and 3B). A detailed description of the RAMP procedure is provided in the Materials and Methods.

One interesting property of ratios combined into a RAMP profile is that knockdowns of a given ribosome synthesis factor generate consistently isomorphic ratio patterns within large margins of silencing levels. As one example of this (Supplementary Figure S3), we carried out several independent transfections to downregulate expression of an essential ribosomal protein. Taken separately, individual precursor ratios varied between experiments, but the similarity of the overall trend in the RAMP profile was retained across a range of knockdown efficiencies (Supplementary Figure S3; see also Supplementary Figure S4 for another example). As illustrated below, another advantage of the RAMP format is that many steps in pre-rRNA maturation can be simultaneously examined, without the need for consulting multiple pictures of blots or graphs. This helps to rigorously evaluate various scenarios and arrive at a model with the best fit for the entire set of data.

### SSU processing factors Rcl1 and Fcf1 are involved in 5′ETS processing and site 2b cleavage in ITS1

The putative mouse SSU synthesis factors Rcl1, Fcf1 and Utp23 chosen for this study have conserved yeast and human homologs that function in 18S rRNA maturation. Using RAMP, we evaluated changes in pre-rRNA processing caused by knockdown of each factor (Figure 2B). Knockdown efficiency after transfection of 3T3 fibroblasts with siGENOME siRNA pools was monitored by quantitative reverse transcriptase-PCR (qRT-PCR) (Supplementary Figure S4A). To ascertain the degree to which the RAMP readout was affected by silencing efficiency, individual RAMP profiles were built for each transfection (Supplementary Figure S4B and C). The overall pattern of changes occurring in Rcl1 and Fcf1 knockdowns showed considerable similarity, and was clearly different from the Utp23 knockdown regardless of depletion levels. Noteworthy, Rcl1 and Fcf1 profiles were not identical, indicating that pre-rRNA processing defects in these cells were specific in each case, and not a general outcome of impaired ribosome synthesis.

The pattern of changes in pre-rRNA ratios in Rcl1 and Fcf1 knockdowns indicated defects in both 5′ETS removal and ITS1 cleavages. The 34S/PTP values significantly increased in both cases (Figure 2B). Processing of 34S pre-rRNA normally begins with cleavage at site A0 (34) generating 29S pre-rRNA, which is then cleaved at site 1 to generate the mature 5′ end of 18S rRNA (Supplementary Figure S2). In the Rcl1 knockdown (Figure 2B), 29S/PTP was elevated, but 20S/PTP decreased, suggesting a reduced efficiency of site 1 cleavage. In agreement with this interpretation, the relative level of the 5′ product of this cleavage, 19S pre-rRNA, was also reduced. In the Fcf1 knockdown, the ratios of 29S, 20S and 19S relative to PTP were all reduced despite a highly elevated 34S/PTP ratio, indicating that site A0 cleavage was suppressed. Consistent with inefficient cleavage at site A0, the product/substrate ratios 29S/34S and 20S/34S were reduced to the same extent in Fcf1 knockdown; in contrast, 20/34S declined stronger than 29S/34S in Rcl1 knockdown (compare lower panels in Figure 2B). In addition to elevated 34S/PTP, Rcl1 and Fcf1 knockdowns displayed a reduction of the 36S/PTP ratio and a significantly increased 34S/36S ratio. Thus, deficiency of either protein had an inhibitory effect on the ITS1 cleavage at site 2b. All these changes were reproducible at different depletion levels (Supplementary Figure S4).

Although Rcl1 and Fcf1 were not expected to function in LSU maturation, RAMP data indicated that their depletion slightly decreased 32S/PTP and 12S/PTP (top panels in Figure 2B). The 32S pre-rRNA is the normal entry point for the production of 5.8S and 28S rRNAs, once ITS1 is completely removed (Figure 1A). In mouse cells, 32S is an abundant and long-lived precursor that is further separated by cleavage at site 4b to generate 12S and 28.5S pre-rRNAs. The modest decrease in 32S/PTP and 12S/PTP is most easily interpreted as a reduced flux of pre-rRNA into the 5.8S/28S pathway because of the inhibitory effects on subunit RNA separation. The 12S/32S ratio, indicative of the state of the downstream processing of 32S pre-rRNA, was not affected in Fcf1 knockdowns. A small decrease in 12S/32S was observed when Rcl1was strongly depleted, but this effect was not reproduced when lower amounts of siRNA were used (Supplementary Figure S4C). We therefore conclude that depletions of these proteins did not significantly affect pre-rRNA maturation in the LSU, in contrast to their effects on 18S processing.

Taken together, these data indicate that (i) mouse Rcl1 and Fcf1 function at distinct 5′ETS processing steps required for the formation of the 5′ end 18S rRNA, consistent with a role in early SSU maturation; (ii) downregulation of Rcl1 or Fcf1 also inhibits cleavage at site 2b, but not at site 2c, in ITS1; (iii) shifting the balance toward cleavage at site 2c has a negligible effect on LSU maturation.

Depletion of yeast Rcl1 was previously reported to inhibit processing at sites A_0_–A_2_, with sites A_2_ and A_1_ affected more strongly than site A_0_ cleavage (19) (yeast pre-rRNA processing is shown in Supplementary Figure S1). Yeast Rcl1 was proposed to act as a nuclease that cleaves pre-rRNA in ITS1 at site A_2_ in ITS1 (22). Yeast Fcf1/Utp24 was suggested to act as a nuclease in processing of site A_1_ at the 5′ end of 18S rRNA and/or site A_2_ (20). Interestingly, yeast Fcf1/Utp24 is also required for the A_0_ cleavage, although this function apparently does not involve its catalytic activity (20). Human RCL1 and UTP24 are required for cleavage at site E/2a in ITS1 (17) and site 1 at the 5′ end of 18S rRNA (24). A decrease of 18S precursors downstream from 30S pre-rRNA in the data shown for depletion of human UTP24 (17) also points to its requirement for site A0 cleavage. In Figure 2B, RAMP clearly shows the difference between steps in 5′ETS processing affected by depletion of the two mouse factors: Rcl1 deficiency more strongly inhibited processing at site 1, while Fcf1 primarily affected cleavage at site A0. The roles of Rcl1 and Fcf1/Utp24 in SSU assembly thus appear to be remarkably conserved across eukaryotic species.

### SSU processing factors Utp23 is required for a late 18S maturation step

Unlike other tested factors, downregulation of Utp23 strongly increased the 18SE/PTP ratio (Figure 2B). Because precursors upstream of 18SE pre-rRNA were decreased, as evidenced by the lowered 20S/PTP and 29S/PTP ratios, the elevated level of 18SE pre-rRNA points to an inhibition of 18SE maturation to 18S rRNA. The decreased 29S and 20S ratios relative to PTP and 34S also suggest an inhibitory effect on earlier processing at sites A0 and 1. All these effects were independent on knockdown efficiency (Supplementary Figure S4). Notably, when Utp23 was strongly depleted, reduction of the 12S/32S ratio was observed (Supplementary Figure S4C), which was more pronounced than for the other SSU factors examined, pointing to possible additional effects of Utp23 depletion on downstream LSU maturation.

Depletion of yeast Utp23 was shown to inhibit A_0_–A_2_ cleavages, but this protein appears to function in a distinct way from Rcl1 and Fcf1/Utp24, by promoting the release of the snR30 snoRNA from preribosomes (23). In human cells, UTP23 is required for 18S rRNA formation, although the previously observed processing effects were reported to be mild because of inefficient depletion (24). The RAMP profile of the mouse homolog's knockdown indicates that normal levels of Utp23 are important for both efficient 5′ETS cleavages and processing of 18SE pre-rRNA, a late step in mammalian 18S rRNA formation absent in yeast. The blocked maturation of 18SE pre-rRNA occurring after cleavage in ITS1 is reminiscent of the effects observed for depletion of late-assembling SSU ribosomal proteins, termed the ‘progression’ group (33). The observed Utp23 depletion phenotype therefore predicts that this protein functions in late SSU maturation, likely downstream from the nucleolar release of nascent pre-40S particles (33). Utp23 knockdown had little effect on 34S/PTP, 36S/PTP and 34/36S ratios of precursors formed by ITS1 processing (Figure 2B), suggesting that neither of the two ITS1 cleavage sites was preferentially affected.

### Impaired LSU assembly affects ITS1 processing at site 2c

We next sought to understand how LSU maturation may affect subunit RNA separation in ITS1. We have shown previously that mouse proteins Pes1 and Nog1 are essential factors in early assembly of the LSU (4,26), similar to their yeast counterparts (35–38). To investigate changes in ITS1 processing conferred by deficiency of Pes1 and Nog1, we used cell lines in which expression of these LSU assembly factors could be downregulated via doxycycline-regulated shRNA (Supplementary Figure S5A).

As shown in Figure 3B, the overall shape of the RAMP profiles for Pes1 and Nog1 knockdowns was drastically different from that of SSU factors (compare with Figure 2B). First, a significant drop in 12S/PTP and 12S/32S, not observed for SSU factor depletions, was immediately notable in both Pes1 and Nog1 knockdowns, consistent with stalled maturation of LSU precursor complexes (4,26). Second, 34S/PTP and 20S/PTP were reduced while 36S/PTP was considerably increased, indicating inhibition of site 2c cleavage. This resulted in a substantial drop in the 34S/36S ratio (Figure 3B, lower panels), opposite to the 34S/36S increase observed in Fcf1 and Rcl1 knockdowns (compare with Figure 2B). The 18SE/PTP ratio was unchanged in the Pes1 knockdown and only marginally decreased in the Nog1 knockdown. Considering the increased 18SE/34S and 18SE/20S values, we conclude that site 2b cleavage was largely unaffected by deficiency in either protein.

Interestingly, while both Nog1 and Pes1 depletions inhibited ITS2 processing as evidenced by the strongly decreased 12S/32S ratio (Figure 3B), RAMP profiles also pointed to their non-identical function in LSU maturation. The 32S/PTP changes for the two proteins were reversed and the Pes1 knockdown more strongly affected the 32S/36S ratio than the Nog1 knockdown. The different roles of these factors were noted in yeast studies where Nop7, the yeast ortholog of mammalian Pes1, was classified as an A_3_ factor, predicted to function earlier than the B factor Nog1 in LSU synthesis (39–41). Unlike Pes1, depletion of Nog1 also shifted 29S/PTP, 19S/PTP and 18SE/PTP down slightly (Figure 3B), indicating a weak inhibitory effect on the SSU processing branch. These observations illustrate that RAMP can be a sensitive tool for revealing nuances in pre-rRNA processing that might not be readily notable through visual inspection of northern blots (Figure 3A).

Based on the results obtained with Pes1 and Nog1 depletions, we conclude that both proteins are required for the efficient cleavage at site 2c, but do not significantly influence site 2b cleavage. Consequently, cells are able to generate the 18S direct precursor, 18SE pre-rRNA, via routes that bypass its typical upstream intermediates 34S and 20S pre-rRNA (Supplementary Figure S2).

### Model for mouse ITS1 processing

**Figure 4.**
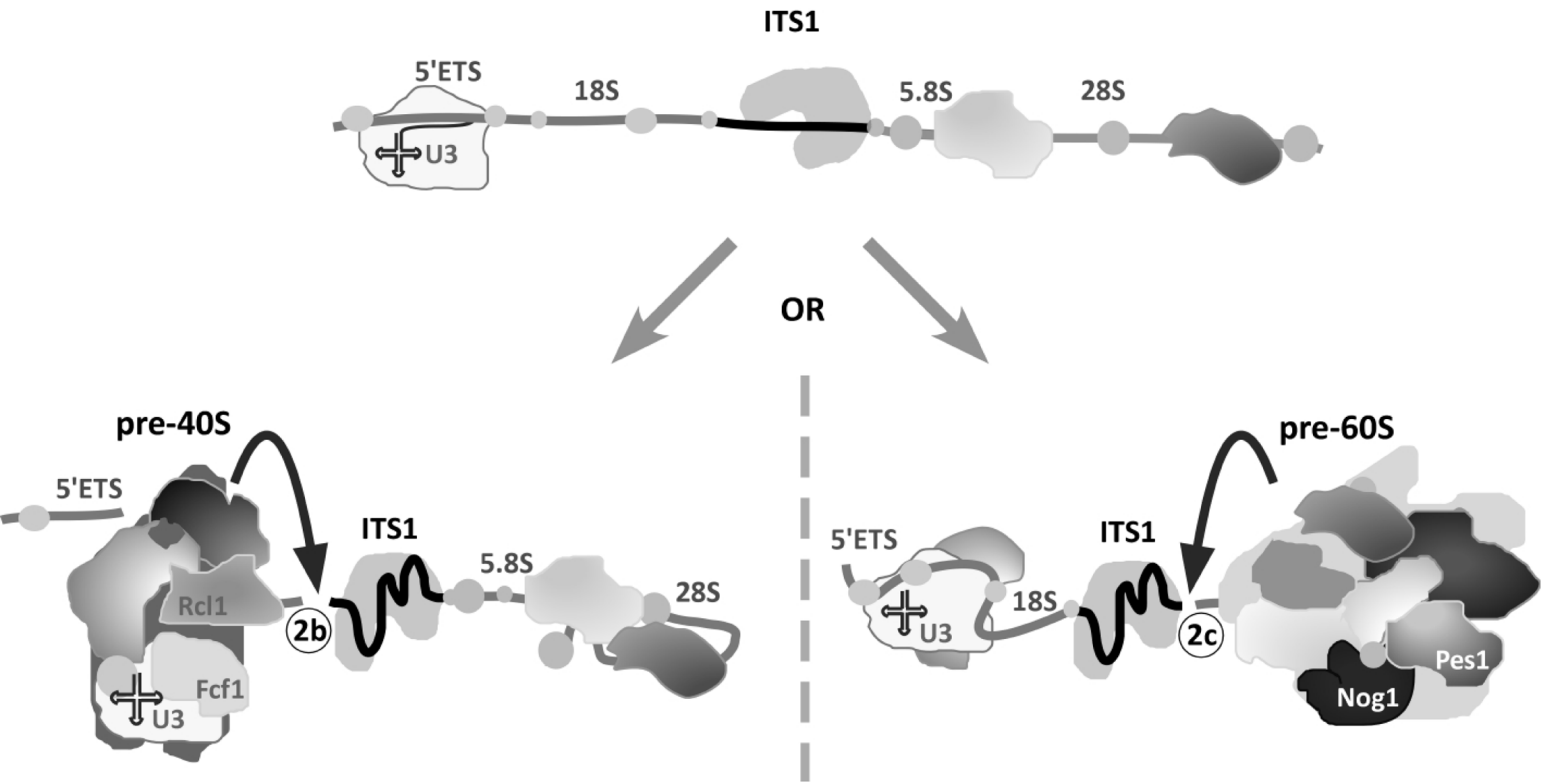
Model for the separation of assembling SSU and LSU through two orthogonal cleavages inside ITS1. The two cleavage sites, 2b and 2c, are located at the opposite ends of the ITS1 sequence. Once a subunit reaches the maturation state permissive for separation, cleavage is triggered at the proximal site, leaving the more slowly assembling subunit attached to the ITS1-protein complex. Assembly within each subunit requires folding of rRNA, binding of ribosomal proteins and coordinated activities of multiple protein assembly factors and snoRNAs (such as U3 snoRNA shown for the SSU).

In the above analysis of the depletions of SSU and LSU factors, we found that one of the two ITS cleavages was preferentially inhibited and this correlated with maturation defects in the subunit proximal to the cleavage site. The simplest hypothesis to account for these observations is that the separating cleavages in pre-rRNA at two ITS1 sites, 2b and 2c, are coordinated with early assembly in the SSU and LSU, respectively (Figure 4). As a result, each subunit remains attached to an ITS1 segment until it reaches the maturation state capable of triggering the separating cleavage on its side of the ITS1. A more general consequence of this arrangement is that a subunit that matures slowly would not prevent the release of the other subunit from the spacer. For example, a delay in the structural transition in pre-18S rRNA required for 5′ETS removal would block cleavage at site 2b, but the primary transcript can still be split at site 2c to release the LSU. Defects in LSU assembly will inhibit cleavage at site 2c, while the SSU can still be released from the pre-rRNA transcript through site 2b cleavage. As we discuss below, this hypothesis can also explain the patterns in pre-rRNA processing observed in a number of previous studies of mammalian ribosomal proteins and ribosome synthesis factors.

### First ITS1 cleavage occurs at site 2c in mouse tissues

**Figure 5.**
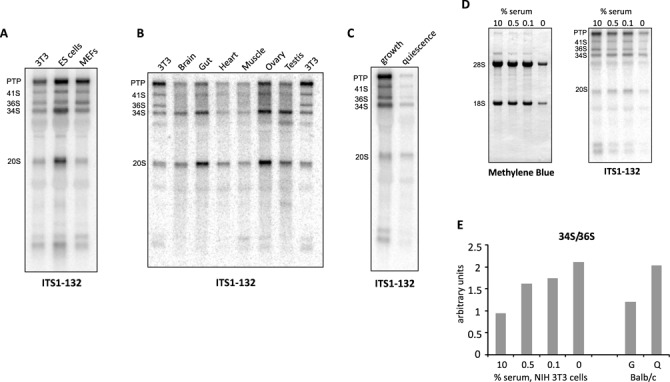
The separation pattern of subunit RNAs depends on cell type and growth conditions. (A) Norther hybridization of RNA extracted from rapidly growing mouse cell lines (NIH 3T3, embryonic stem cells and primary embryonic fibroblasts) with an ITS1 probe that detects precursors formed by both 2b and 2c cleavages. (B) Hybridization of RNA from various mouse tissues in comparison with 3T3 cells. Methylene blue staining of rRNA on the same membrane and additional hybridizations can be found in Supplementary Figure S6. (C) Hybridization of RNA isolated from rapidly growing and quiescent Balb/c cells. (D) RNA isolated from NIH 3T3 cells cultured in medium containing 10% calf serum or with reduced serum concentrations for 18 h. RNA blotted on a membrane was stained with methylene blue and hybridized with the indicated ITS1 probe. (E) Quantification of the 34S/36S ratio on blots shown in panels C and D.

Two rRNA precursors, 34S and 36S, formed as a result of two alternative orders of ITS1 cleavages (Figure 1A), are present at comparable levels in mouse 3T3 cells (Figure 1C). In contrast, primary human cells and many human cell lines display little 36S pre-rRNA under normal conditions (11,15,16). To investigate the nature of this difference further, we assayed steady-state levels of various pre-rRNAs in other types of mouse cells. In cultures of embryonic stem cells and primary embryonic fibroblasts, 36S pre-rRNA was present together with other major rRNA precursors (Figure 5A). However, when we analyzed total RNA isolated from various mouse tissues, we found that 36S pre-rRNA was less abundant, with a moderately elevated level in some organs, such as the ovary (Figure 5B). Thus, although splitting of the mouse pre-rRNA transcript could be initiated at site 2b and 2c in cultured cells, initial cleavage at site 2c is prevalent in many tissues inside the organism.

Because most cells in adult tissues are quiescent, we asked whether the increased utilization of site 2b in cell lines may be related to the fact that these cells are actively proliferating. To address this, we induced cells to exit the cell cycle and enter a quiescent state. Contact-inhibited Balb/c cells were made quiescent by an established protocol (42). In another set of experiments, we transferred NIH 3T3 cells from their normal growth medium containing 10% serum into media with 0.5%, 0.1% and 0% serum for 18 h, while control cells remained in 10% serum. We assayed RNAs extracted from these cells by northern hybridizations (Figure 5C and D). As expected, levels of all pre-rRNAs declined when serum concentration was reduced because of diminished Pol I transcription (compare PTP levels in Figure 5C and D). Quantifying the northern data revealed that, in all cases, the 34S/36S ratio increased when cells were no longer rapidly dividing (Figure 5E), and more closely resembled the cleavage pattern observed in RNA isolated from adult mouse tissues as well as human cells. Thus, growth factor signaling that promotes fast cell growth and division also leads to an increase in the split of pre-rRNA through site 2b.

## DISCUSSION

### Interrogating pre-rRNA processing with RAMP

Reliable identification of the rRNA maturation steps affected by deficiency of a ribosome assembly factor can be non-trivial, given that altered steady-state levels of pre-rRNAs often reflect changes in multiple steps of the pathway and also vary depending on the extent of the factor's depletion. In this report, we show that evaluating RAMP profiles provides a sensitive way to resolve processing steps affected by knockdowns of ribosome synthesis factors in mammalian cells. The analysis workflow described here also helps to deal with routine technical hurdles, such as variations in transfections, unequal sample loading and variable probe hybridization efficiencies. We suggest that RAMP can improve functional annotation and characterization of ribosome synthesis factors and may offer a convenient framework to enable comparison of data by researchers working in different species or with different cell types. RAMP may be especially useful in studies of ribosome biogenesis when only subtle changes in pre-rRNA processing are observed. This is, for example, often the case in ribosomopathies, disorders associated with the partial loss of function in genes required for ribosome synthesis (43,44).

### Common features of ITS1 processing in human and mouse cells

A key event in processing of the pre-rRNA transcript is the separation of subunit RNAs through endonucleolytic cleavages in ITS1. ITS1 processing has been recently examined in human cells (15–17). In this work, we sought to characterize cleavage sites in mouse ITS1 in order to gain a better understanding of the conservation of mammalian pre-rRNA processing features. By using RAMP to examine ratios of pre-rRNAs formed by ITS1 cleavages, we found that the 18S-proximal cleavage at site 2b was suppressed by depletion of proteins Rcl1 and Fcf1 involved in early SSU maturation, but not by depletion of Utp23 or LSU assembly factors. The effects of Rcl1 and Fcf1 depletion in mouse cells agree well with results from studies of human RCL1 and FCF1/UTP24 (17,24), providing a strong argument for a link between 5′ETS processing and 18S-proximal cleavage in ITS1 (human site E/2a, mouse site 2b). In another study of human SSU ribosomal proteins (33), depletion of any of the 16 early assembling ‘initiation’ ribosomal proteins resulted in a strong reduction of the 18S-E pre-rRNA. It was suggested that a minimal folding of the body and the head of the SSU is a prerequisite for cleavage in ITS1 (33). Importantly, lack of ribosomal proteins from the initiation group did not prevent human 30S pre-rRNA (equivalent to mouse 34S) from being generated, indicating that only the cleavage at the 5′ side of ITS1 was inhibited, but not at the 3′-terminal site, equivalent to site 2c in the mouse.

Depletion of LSU assembly factors Pes1 or Nog1 (4,25,26,45) caused a series of changes consistent with a strong inhibition of cleavage at the 3′-terminal ITS1 site 2c, but not the 5′-terminal ITS1 cleavage at site 2b. The selective inhibition of cleavage at human site 2, equivalent to mouse 2c, was also observed in knockdowns of LSU assembly factors (16,17), as well as certain LSU ribosomal proteins, such as RPL26 (46). In our previous work, metabolic labeling in cells expressing dominant negative mutants of the LSU assembly factors Bop1 and Pes1 showed that robust production of 18S rRNA in mouse 3T3 cells can take place in the absence of 28S rRNA synthesis (4,27,47). In another study, depletion of several human LSU ribosomal proteins (48) allowed both 18S rRNA and its direct precursor 18S-E pre-rRNA to form despite a severe reduction in 28S rRNA synthesis. Finally, cleavage at ∼55 nt downstream of the 3′ end of 18S rRNA in mouse cells was reported in transcripts arising from transfected constructs lacking the 28S rRNA coding sequence (32), indicating that cleavage at the site that we have now mapped as 2b can occur independently from LSU assembly.

The data from previous human and mouse studies thus appear to be consistent with the model in which early assembly events in each ribosomal subunit control a proximal cleavage in ITS1 (Figure 4). Notably, this mode of splitting the SSU and LSU precursors does not preclude the existence of factors that may be involved in both ITS1 cleavages. One possible role for such factors could be, for example, to prevent subunit separation from ITS1 until a defined maturation state is attained.

### ITS1 processing in yeast and mammalian cells

Considerable differences exist between higher and lower eukaryotes in ITS1 composition and structure. Mammalian ITS1 sequences have a much higher G+C content than yeast (70.1% in mouse versus 35.2% in yeast) and are typically 2–3 times longer (49). In *S. cerevisiae*, two endonucleolytic cleavage sites, A_2_ and A_3_, exist in ITS1 ((3,50), Supplementary Figure S1B). During rapid growth, 70–80% Pol I transcripts in yeast are cleaved at site A_2_ cotranscriptionally (14). Suboptimal growth conditions, as well as a variety of mutations affecting either SSU or LSU synthesis, induce a shift to post-transcriptional separation of subunit RNAs (13). As a result, the accumulating 35S pre-rRNA can be cleaved at site A_3_ in the absence of upstream cleavages at sites A_0_, A_1_ and A_2_, generating 23S RNA, an aberrant intermediate that does not appear to be a substrate for 18S rRNA formation (50). Consistent with the inhibition of cotranscriptional cleavage, yeast Rcl1, Utp23 and Fcf1/Utp24 were found to be essential for processing at sites A_0_–A_2_ (19–23). Depletion of the LSU assembly factors, such as Nog1 and Nop7, the yeast ortholog of Pes1, also inhibited contranscriptional cleavage at site A_2_, resulting in 35S pre-rRNA accumulation (35,38,51,52).

In contrast to yeast, splitting of the mouse transcript through either of the two sites located within ITS1 generates mainstream precursors that mature to 18S and 5.8S/28S rRNAs (Figure 1A, Supplementary Figure S2). As mentioned above, deficiencies in mouse Rcl1 and Fcf1, both of which act early in 18S rRNA maturation, inhibit ITS1 cleavage at site 2b, lending support to the notion that the 5′-terminal ITS1 site (mouse site 2b, human site E/2a) is analogous to site A_2_ in budding yeast (17). However, defects in early LSU assembly in mouse and human cells only inhibit strongly cleavage in the 3′ region of ITS1. The separation of subunit RNAs in mammals thus appears to involve two orthogonal cleavages in ITS1, a distinct strategy from that in budding yeast.

### Influence of cell physiology on subunit RNA separation

Early studies of pre-rRNA in higher eukaryotes led to the concept of alternative pre-rRNA processing pathways (53–56). For instance, in human cells, 36S pre-rRNA has been considered to be a product of the ‘minor’ pathway since this precursor is typically present in trace amounts (15,16,56), whereas mouse cell lines display greater levels of 36S pre-rRNA (Figure 5A). Much of the accumulated evidence suggests that the difference between processing pathways is due to variations in the temporal order of early pre-rRNA cleavages rather than distinct underlying mechanisms (1,34,55). What could account for the persistency of the pre-rRNA processing patterns in different cells? One interesting implication of our model (Figure 4) is that the previously described ‘processing pathways’ can be explained by kinetic competition between two ribosomal subunits during their initial assembly on the common pre-rRNA transcript. If the SSU is first to reach the state competent for release, cleavage through site 2b will generate 18SE and 36S pre-rRNAs; if the LSU is first to mature, 34S, 29S or 20S pre-rRNAs are generated on the 18S side and 32.5S pre-rRNA on the 5.8S/28S side (Figure 1A). Any factors that preferentially affect assembly of one of the subunits will thus change the appearance of which pathway predominates. For instance, a slowdown in LSU assembly will result in a higher percentage of 18S precursors completing 5′ETS processing and separating from the transcript through site 2b cleavage before cleavage at site 2c takes place (Figure 1A, left arrow). This shift in the order of ITS1 cleavages will manifest as an increase in the ratio of 36S pre-rRNA to 34S, 29S and 20S pre-rRNAs (note that in kinetic analysis, this would appear as increased half-life of 36S pre-rRNA).

The lower steady-state level of 36S pre-rRNA in many adult mouse tissues, as compared with rapidly proliferating mouse cell lines (Figure 5B), indicates that like in humans (17), the first cleavage of the mouse transcript typically occurs on the LSU side in the organism. The observed changes in the ITS1 cleavage pattern in response to growth factor stimulation (Figure 5C–E) further imply that conditions favoring growth may be capable of promoting early SSU assembly to a larger extent than that of LSU. The yeast-like mode of splitting the pre-rRNA through cleavage at the 5′ site in ITS1 could represent an adaptation that helps to increase rRNA production in rapidly proliferating mouse cells. An alternative, albeit not mutually exclusive, explanation is that the complicated LSU assembly may require more resources than SSU assembly, and consequently lags behind during rapid ribosome synthesis. Regardless, the precursor ratios indicating the order of ITS1 cleavages can be a sensitive indicator of conditions that differentially affect early assembly of the two subunits. Apart from experimentally induced factor depletion, such conditions might arise from insufficient cellular levels of certain ribosomal proteins and assembly factors required for early assembly steps as well as imbalanced expression of the myriad components of the ribosome synthesis machinery. Considering that extensive genome rearrangements is a hallmark of cancer cells, the possibility that the ratios of intermediates formed by ITS1 cleavages might serve as a marker for pathological shifts in gene expression merits further investigation.

## SUPPLEMENTARY DATA

Supplementary Data are available at NAR Online.

SUPPLEMENTARY DATA
