# Supplementary material for: Two orthogonal cleavages separate subunit RNAs in mouse ribosome biogenesis
Source: Nucleic Acids Res. 2014 Sep 4;42(17):11180–91. doi: 10.1093/nar/gku787 (PMC4176171; doi:10.1093/nar/gku787)
Supplement: SUPPLEMENTARY DATA [file supp_42_17_11180__index.html]

Two orthogonal cleavages separate subunit RNAs in mouse ribosome biogenesis — Two orthogonal cleavages separate subunit RNAs in mouse ribosome biogenesis — SUPPLEMENTARY DATA 

# Two orthogonal cleavages separate subunit RNAs in mouse ribosome biogenesis

## SUPPLEMENTARY DATA

**Files in this Data Supplement:**

- SUPPLEMENTARY DATA
