# Supplementary material for: Two orthogonal cleavages separate subunit RNAs in mouse ribosome biogenesis
Source: Nucleic Acids Res. 2014 Sep 4;42(17):11180–91. doi: 10.1093/nar/gku787 (PMC4176171; doi:10.1093/nar/gku787)
Supplement: SUPPLEMENTARY DATA [file supp_gku787_nar-00753-y-2014-File007.pdf]

SUPPLEMENTAL DATA

Table S1. Oligonucleotide probes and PCR primers used in this study.

Oligo	Sequence (5' to 3')
5'-ETS-end	ggacagagagcgcgagagag
ITS1-29	acgccgccgctcctccacagtctcccgtt
ITS1-54	acacacaagacggggagagcgggccacgcc
ITS1-85	acccgggacgcacgcgcctc
ITS1-184	aggggaaatcggaggcggtc
ITS1-132	ttctctcacctcactccagacacctcgctccaca
ITS2-32	acccaccgcagcgggtgacgcgattgatcg
qRPL23-for2	atccagcagtggtaattcgaca
qRPL23-rev2	cccctgcgttatcttcaaagt
qRPL17-for2	atggttcgctactctcttgacc
qRPL17-rev2	agtgaacacgaaggtttgacc
qRcl1-for2	aatcaaccaaacaggaacaacct
qRcl1-rev2	cgcagaacgatttttaatgggtg
qUtp23-for1	aaacacgccaagaagcatctc
qUtp23-rev1	gtctctcccataaggtagcgg
qFcf1-for1	tgaccgtgacctcaaacgaag
qFcf1-rev1	ggggctccataatcatctggc
qPes1-F1	tgccaccaattatatcacccga
qPes1-R1	ggagcttccgaacaaacacc
qNog1-F1	acttcaagaagattacggtggtg
qNog1-R1	cgttggagtctttcgttgagt

Primers for qPCR were designed via PrimerBank (http://pga.mgh.harvard.edu/primerbank/index.html).

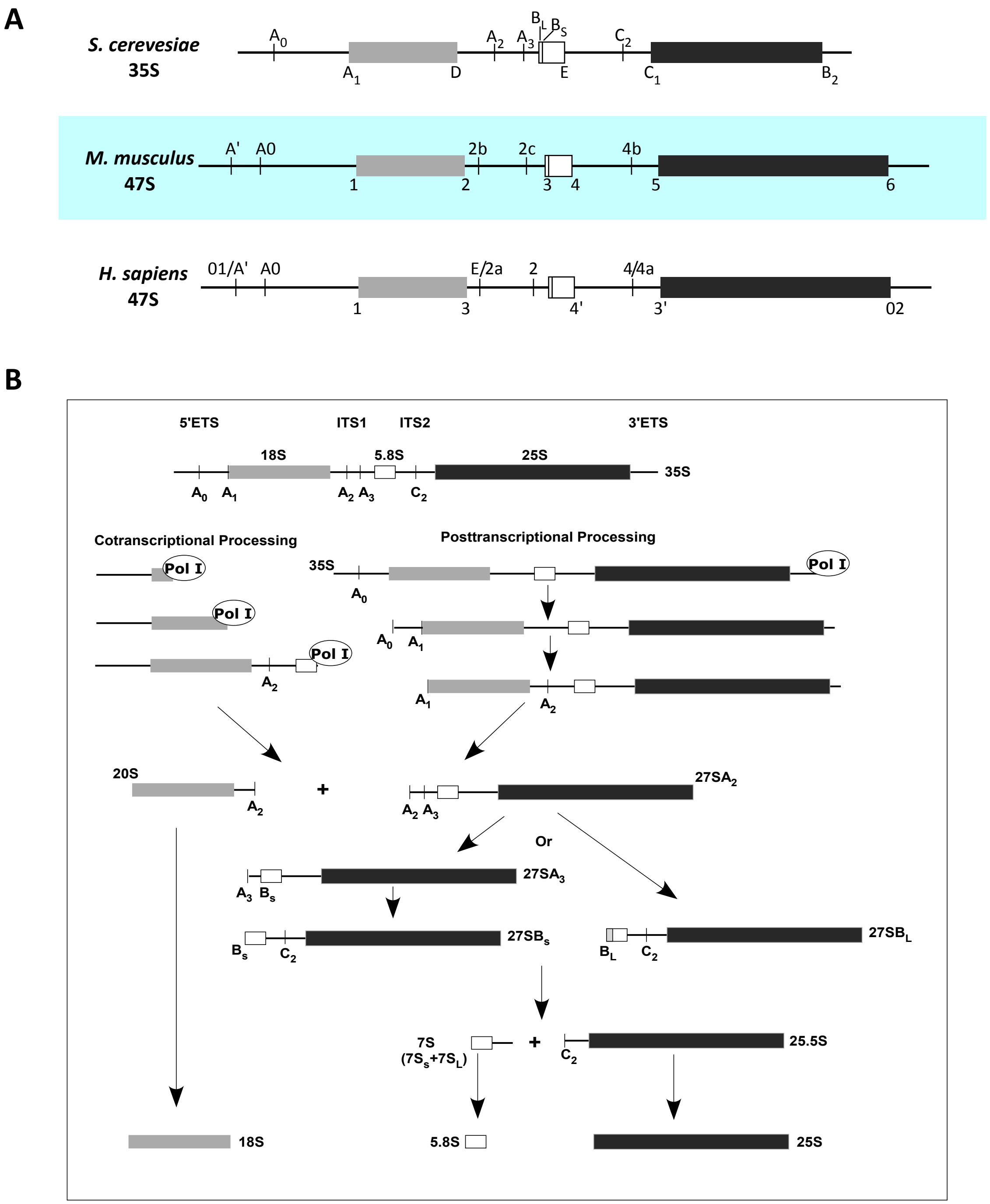

Figure S1. Pre-rRNA in different eukaryotic species. (**A**) Schematic structures of primary Pol I transcripts in yeast, mouse and human. Main processing sites are shown. (**B**) Major intermediates formed in cotranscriptional and posttranscriptional processing pathways in *S. cerevisiae*.

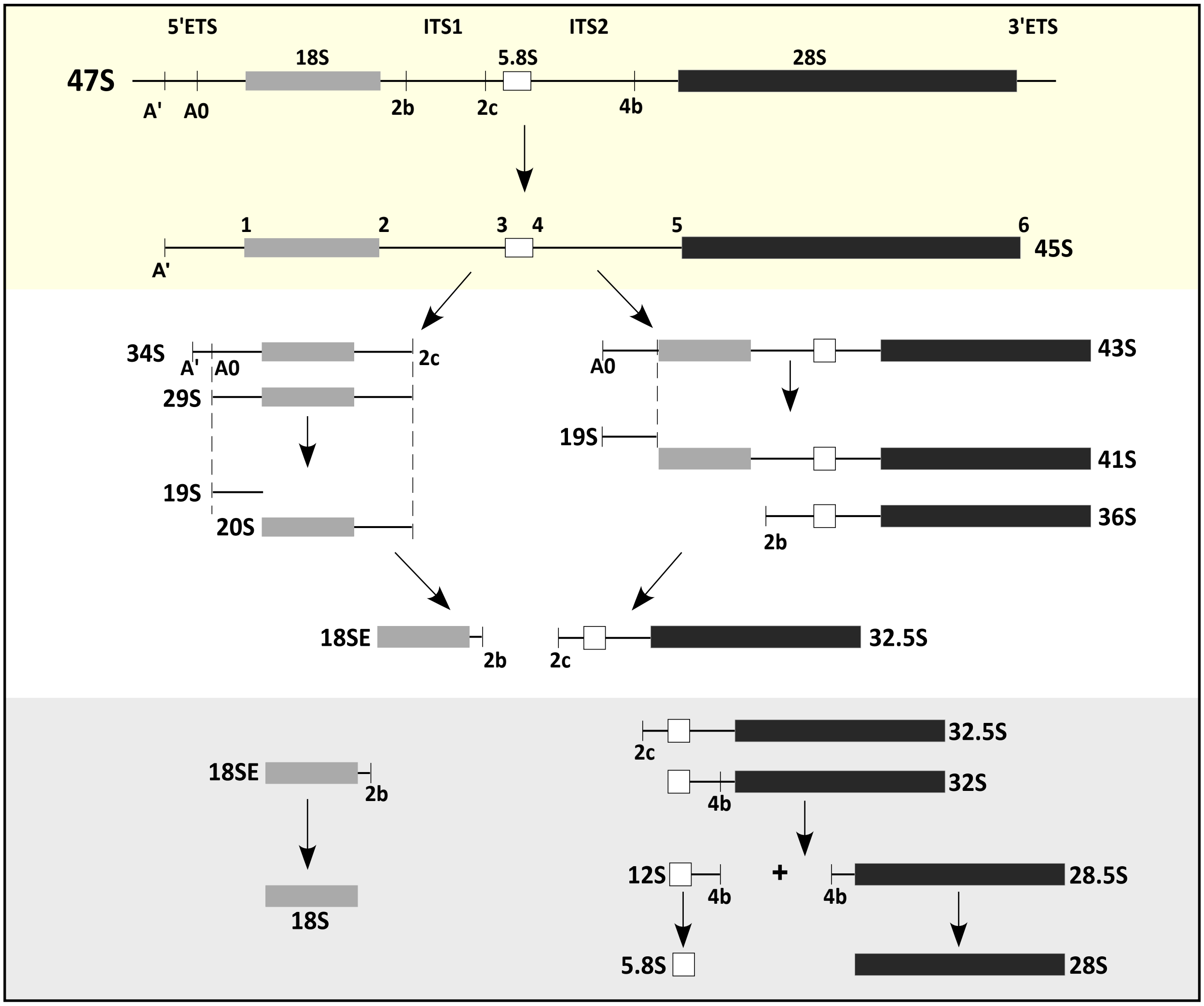

Figure S2. Processing of mouse pre-rRNA. The full-length polymerase I transcript, 47S pre-rRNA, is rapidly cleaved at sites A' and 6 after its synthesis, releasing 45S pre-rRNA. At subsequent processing steps, cleavages at sites in 5'ETS and ITS1 can occur in a variable order. The two commonly observed sequences of cleavage events are presented in the middle part of the diagram. Removal of 5'ETS and the split in ITS1 start the independent maturation of the rRNAs in the two ribosomal subunits.

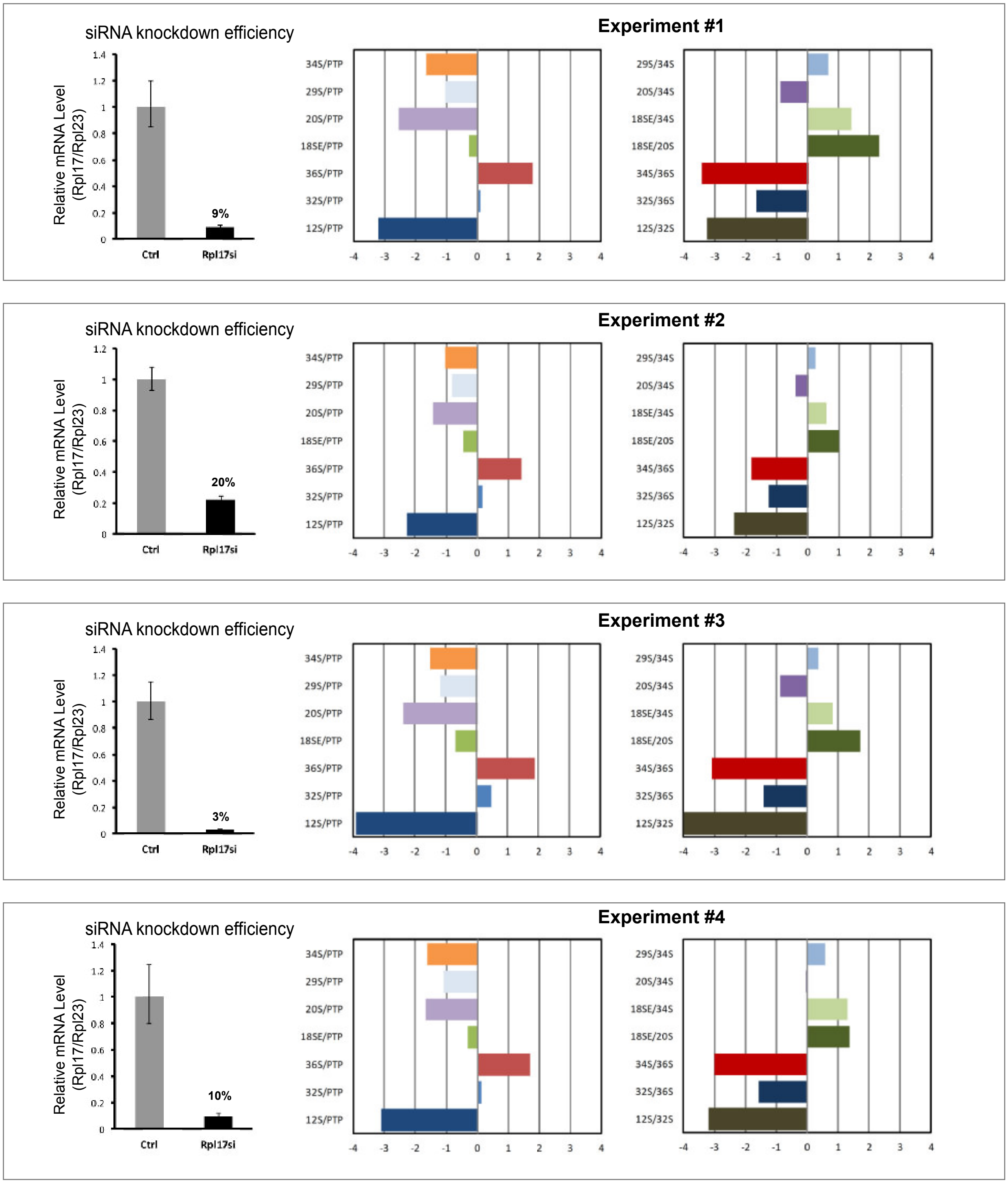

Figure S3. Different silencing efficiencies result in a similar RAMP profile. Four independent transfections of 3T3 cells with siRNA targeting ribosomal protein Rpl17 are shown. Knockdown efficiency in each experiment was determined by qRT-PCR as the Rpl17 mRNA level relative to that of an unrelated ribosomal protein Rpl23. Rpl17/Rpl23 mRNA ratios are normalized to the average ratio in cells transfected with a nontargeting siRNA pool (Ctrl); error bars, SD; n=4. The two RAMP readouts shown for each experiment represent $\log_2$ values for pre-rRNA/PTP ratios (left) and ratios between selected precursors (right). Hybridization probes used to obtain the RAMP data are the same as in the main figures.

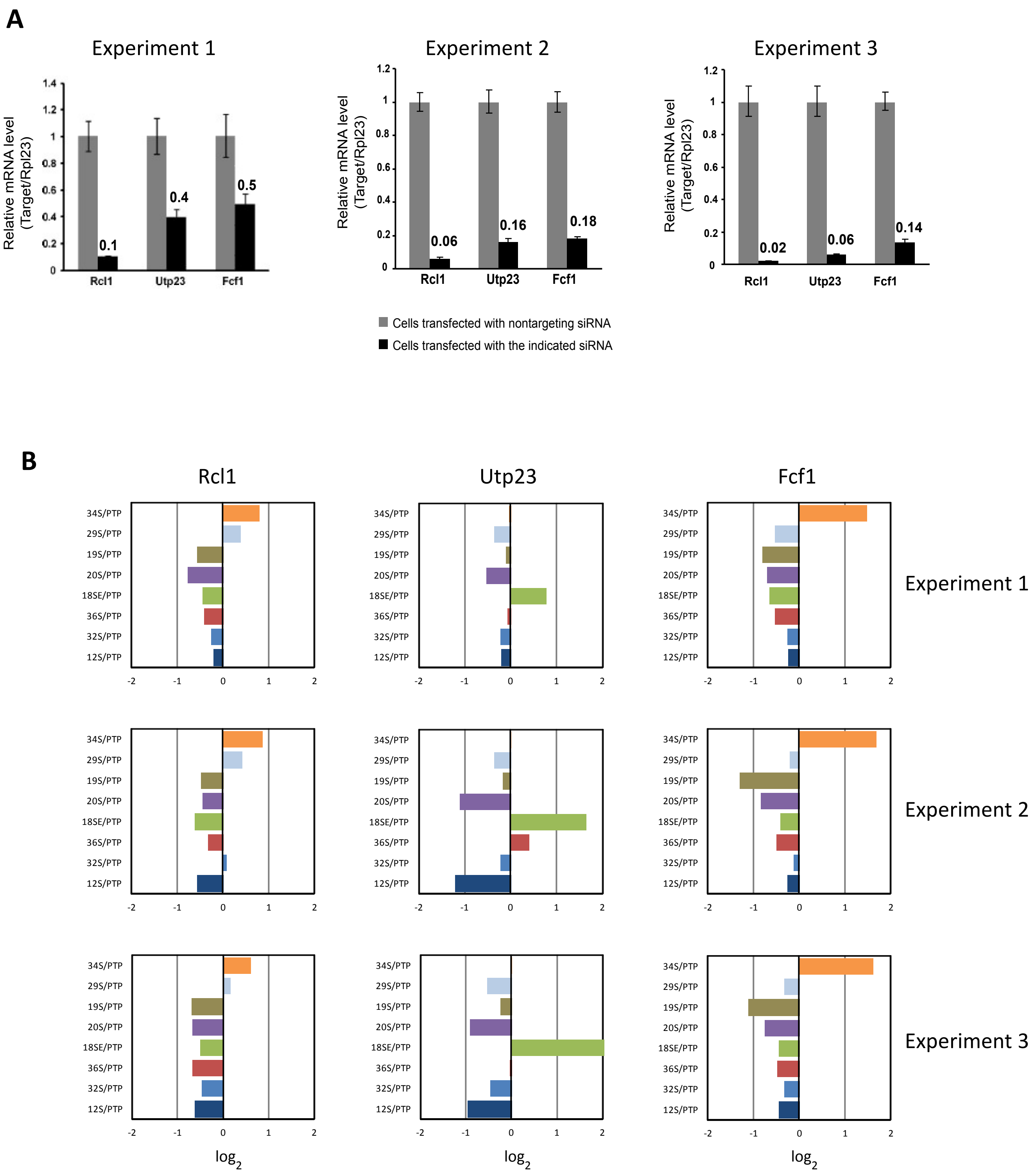
A
Experiment 1
Experiment 2
Experiment 3
Relative mRNA level (Target/Rpl23)
Rcl1
Utp23
Fcf1
0.1
0.4
0.5
0.06
0.16
0.18
0.02
0.06
0.14
Cells transfected with nontargeting siRNA
Cells transfected with the indicated siRNA
B
Rcl1
Utp23
Fcf1
34S/PTP
29S/PTP
19S/PTP
20S/PTP
18SE/PTP
36S/PTP
32S/PTP
12S/PTP
Experiment 1
Experiment 2
Experiment 3
log2

C

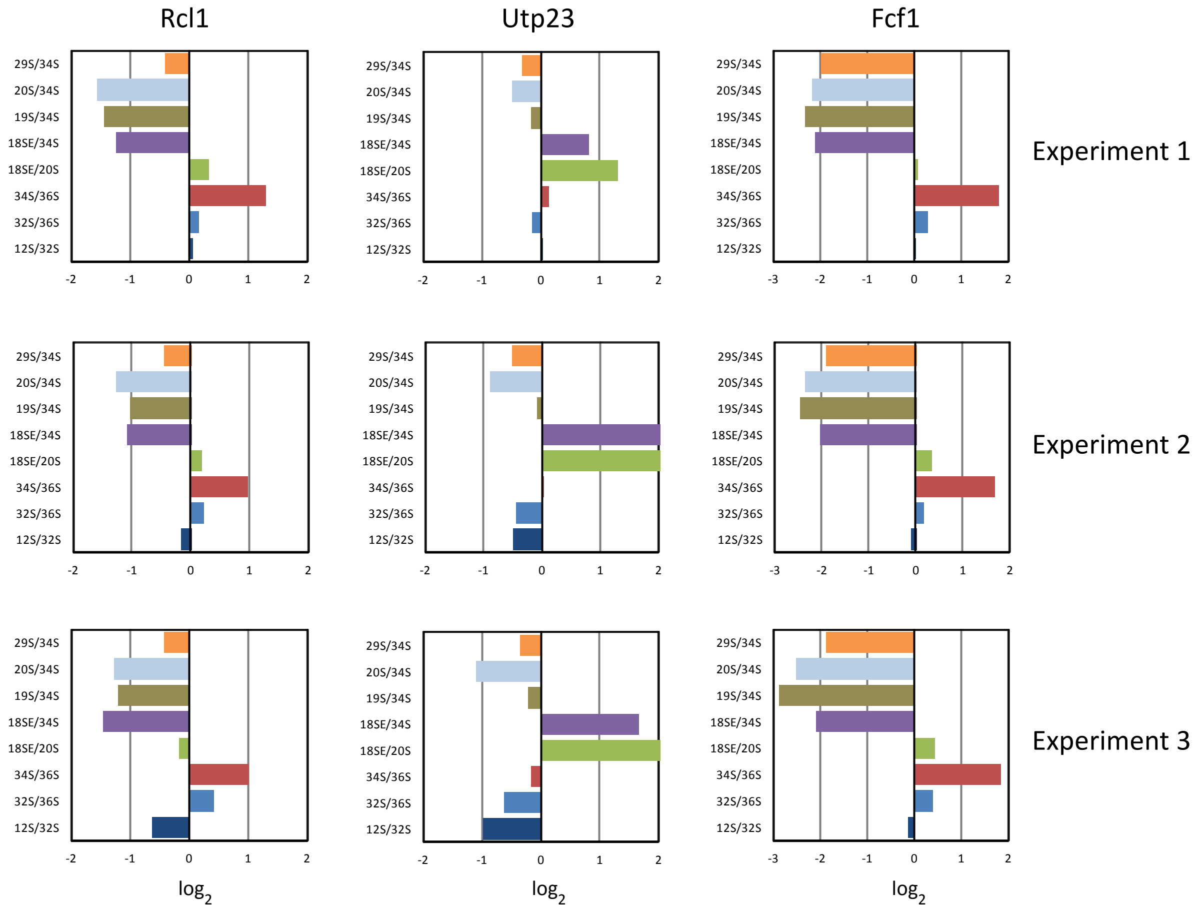

Figure S4. RAMP profiles for three independent transfections of 3T3 cells with siRNA targeting Rcl1, Utp23 and Fcf1. (**A**) Knock-down efficiency in each transfection experiment was determined by qRT-PCR as the ratio of the target gene's mRNA to Rpl23 mRNA. Target/Rpl23 mRNA ratios were normalized to the average ratio in cells transfected with a nontargeting siRNA pool (Ctrl); error bars, SD; n=4. (**B, C**) RAMP profiles for each transfection experiment. Hybridization probes used to obtain the RAMP data are the same as in Figure 2.

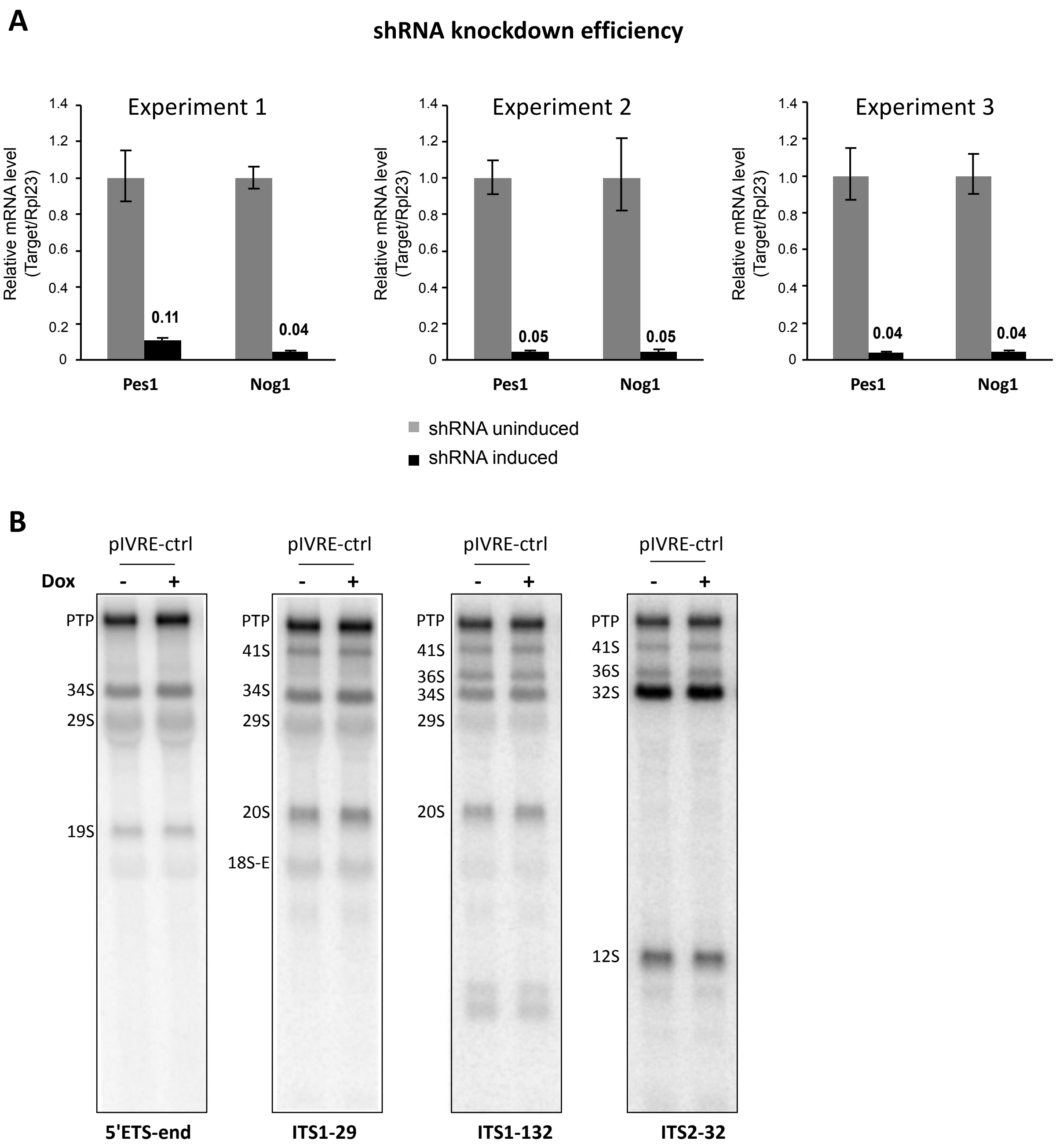

Figure S5. Controls for knockdown experiments in cells stably transfected with pIVRE-shRNA constructs. (**A**) qRT-PCR data used to estimate knockdown efficiency of the target genes. Ribosomal protein Rpl23 mRNA was used as a reference. The target/Rpl23 mRNA ratios in doxycycline-induced cells are normalized to uninduced cells. Error bars indicate SD; n=4. (**B**) Northern hybridizations with RNA isolated from cells transfected with the pIVRE vector lacking an shRNA cassette. No detectable changes in pre-rRNAs were observed after incubation of cells with doxycycline.

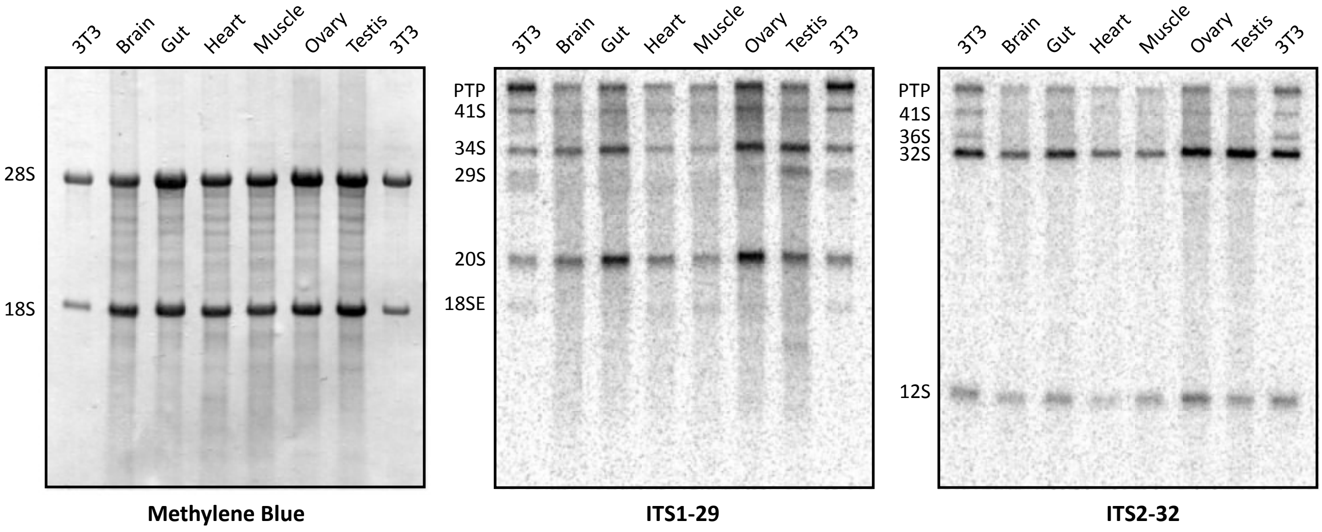

Figure S6. Methylene blue staining of total RNA for the northern hybridization shown in Fig. 5B and additional hybridizations of the same membrane with the indicated probes.
